# Editorial Brevities

**Published:** 1888-04

**Authors:** 


### EDITORIALS AND MISCELLANEOUS.

### EDITORIAL BREVITIES.

CALLS.—Dr. T L. Lallerstedt, of Panola, Ga., and Dr. A. L. Barry, of Ringgold, Ga., called at our sanctum a few days ago. Dr. L. is devoted to gelsemium as the remedy par-excellence in many affections, while Dr. B. is equally strong in his advocacy of veratrum a superior even to gelsemium. Their controversy upon the comparative merits of these two drugs was decidedly animated and spicy.

PROF. POWELL's charge to the Medical and Dental graduating classes at the Southern Medical College Commencement will be found interesting and profitable reading matter in this number, containing both thought and sentiment—eloquently expressed. The moral as well as the intellectual training of students is kept in view in this institution. It is indeed an excellent school in all ats departments, and may now be said to have safely emerged from its youthful or incipient stage into the full vigor of manhood and completeness. W.

COL. NESBIT's SPEECH.—The address of Col. Nesbit at the late commencement of the Southern Medical College will be found in the present number of our journal. It is an able address, and is worthy to be carefully read by every member of the profession as containing important information upon many points in which both the profession and the public are interested. Among the points worthy of note are the success and the growing arrogance of all species of quackery. The indifference of the law-making power and the public at large, especially in Georgia, to the claims of the profession ; the want of recognition, and the want of protection to the health of the people, etc. We cannot refer in detail to these and other very interesting facts brought out in this speech. The profession should read and ponder well this excellent address. Col. Nesbit's eulogy upon the true physician, as illustrated by the soldier in one of the grandest battles of the late war, is the most eloquent and touching tribute to the medical fraternity that we have ever heard, and may well cause a blush of pride and pleasure to mantle the cheek of every true member of our noble profession.

Col. Nesbit's remarks on delivering the prizes were beautiful, but are crowded out of the present journal for want of space.

THE MEDICAL ASSOCIATION OF GEORGIA will meet in Rome on the third Wednesday in April. Let all go who can, and contribute by their presence to the number and to the social enjoyment of the occasion. And let every one who can write a creditable paper (and Georgia has many such) do so for that association. The object of these associations is two-fold—the one to promote the brotherhood, the other to advance the knowledge of the members.

THE ARKANSAS MEDICAL ASSOCIATION convenes at Little Rock on the 25th inst. It will constitute its 13th annual meeting.

THERE are 170 female physicians practicing in the city of New York.

CHLOROFORM and ether are said to be antagonistic to cocaine, and should be inhaled in cases of poisoning by the latter.

A NATIONAL DENTAL MUSEUM is now being discussed as among the probabilities of the early future.

THE MISSOURI STATE MEDICAL ASSOCIATION meets at Kansas City on April 17th. A very large attendance is expected.

I. PHILLIPS, dealer in Surgical Instruments and physicians' supplies, keeps everything that a doctor wants. He will be present, we learn, at the Medical Association of Georgia with an interesting outfit of surgical instruments. Call and see him.

DOG DOCTORS.—There are doctors in New York who devote themselves to treating sick dogs and do a more profitable business than many who practice upon the genus homo.

THE SAMPLE COPY MAN.—Medical journalists have ascertained that less than one-third of the doctors subscribe for medical journals. A majority, it is believed, do not read the journals—while the fact that so many sample copies are called for without even the price of a single copy, has led to the belief that a considerable number get all their reading in that way; a one cent postal card serving to secure a copy of a journal worth 20 to 50c and postage paid. Of course no honorable man in the profession will act in this way.

JOHN B. DANIEL'S Drug Store is just opposite the entrance to the Union Passenger Depot in Atlanta. When you enter the city to look around and make your purchases you will find this a good establishment and very convenient. Physicians' supplies a specialty. A very large lot of surgical instruments on hand at exceedingly low figures. Orders for drugs or surgical instruments promptly and satisfactorily filled.

RECEIPTED—To January 1st, 1888.—Drs. G. W. Boykin, J. N. Ryan, I. H. McMullan, T. R. Mitchell, W. B. Rushin, Lib. Surg. General, E. Wheeler, Van B. Watson, Jno. H. Young, L. B. Bouchelle, J. E. Rodgers, C. S. Priestly, R. H. Edwards, J. T. Young, C. C. Hammond, W. W. Mahan, to June; L. Allison, T. S. Fox, to November; B. F. Duke, to July; J. T. Sego, to April; W. T. Foute, G. L. Mills, to July; N. B. Warren, R. C. McCann, J. M. Bosworth, D. S. Cheatham, W. M. Peacock, to March; E. A. Anderson, to July.

TO JANUARY 1889.—J. Dilworth, E. H. Parham, J. M. Bently, M. D. Miles.

FOR 1886.—G. M. Luster.

JACOBS' PHARMACY.—This large establishment is located on Marietta, corner of Peachtree street. They keep a large stock and sell at low prices. They are polite and active business men, and do a good deal of manufacturing. See their advertisement and price list an advertising page 10.

SANDERS & SONS' EUCALYPTI EXTRACT (*Eucalyptol*).—Apply to Dr. Sanders, Dillon, Iowa, for gratis supplied reports on cures effected at the clinics of the Universities of Bonn and Griefswald.

